# Spatiotemporal patterns of leaf nutrients of wild apples in a wild fruit forest plot in the Ili Valley, China

**DOI:** 10.1186/s12870-024-05417-6

**Published:** 2024-07-18

**Authors:** Meng-Ting Wang, Zhi-Fang Xue, Ye Tao, Zi-Han Kan, Xiao-Bing Zhou, Hui-Liang Liu, Yuan-Ming Zhang

**Affiliations:** 1 State Key Laboratory of Desert and Oasis Ecology, Key Laboratory of Ecological Safety and Sustainable Development in Arid Lands, Xinjiang Institute of Ecology and Geography, Chinese Academy of Sciences, Urumqi, Xinjiang 830011 China; 2 Xinjiang Field Scientific Observation Research Station of Tianshan Wild Fruit Forest Ecosystem, Xinyuan, Xinjiang 844900 China; 3 Xinjiang Key Laboratory of Biodiversity Conservation and Application in Arid Lands, Xinjiang Institute of Ecology and Geography, Chinese Academy of Sciences, Urumqi, Xinjiang 830011 China; 4 College of Life Science, Shihezi University, Shihezi, Xinjiang 832003 China

**Keywords:** *Malus sieversii*, Leaf stoichiometry, Nutrient resorption, Seasonal variation, Spatial distribution pattern, Influencing factor

## Abstract

*Malus sieversii*, commonly known as wild apples, represents a Tertiary relict plant species and serves as the progenitor of globally cultivated apple varieties. Unfortunately, wild apple populations are facing significant degradation in localized areas due to a myriad of factors. To gain a comprehensive understanding of the nutrient status and spatiotemporal variations of *M. sieversii*, green leaves were collected in May and July, and the fallen leaves were collected in October. The concentrations of leaf nitrogen (N), phosphorus (P), and potassium (K) were measured, and the stoichiometric ratios as well as nutrient resorption efficiencies were calculated. The study also explored the relative contributions of soil, topographic, and biotic factors to the variation in nutrient traits. The results indicate that as the growing period progressed, the concentrations of N and P in the leaves significantly decreased (*P* < 0.05), and the concentration of K in October was significantly lower than in May and July. Throughout plant growth, leaf N–P and N–K exhibited hyperallometric relationships, while P–K showed an isometric relationship. Resorption efficiency followed the order of N < P < K (*P* < 0.05), with all three ratios being less than 1; this indicates that the order of nutrient limitation is K > P > N. The resorption efficiencies were mainly regulated by nutrient concentrations in fallen leaves. A robust spatial dependence was observed in leaf nutrient concentrations during all periods (70.1–97.9% for structural variation), highlighting that structural variation, rather than random factors, dominated the spatial variation. Nutrient resorption efficiencies (NRE, PRE, and KRE) displayed moderate structural variation (30.2–66.8%). The spatial patterns of nutrient traits varied across growth periods, indicating they are influenced by multifactorial elements (in which, soil property showed the highest influence). In conclusion, wild apples manifested differentiated spatiotemporal variability and influencing factors across various leaf nutrient traits. These results provide crucial insights into the spatiotemporal patterns and influencing factors of leaf nutrient traits of *M. sieversii* at the permanent plot scale for the first time. This work is of great significance for the ecosystem restoration and sustainable management of degrading wild fruit forests.

**Supplementary Information:**

The online version contains supplementary material available at 10.1186/s12870-024-05417-6.

## Introduction

Ecological stoichiometry mainly investigates the interrelationships and balance of essential nutrient elements (such as carbon (C), nitrogen (N), phosphorus (P), and potassium (K)) crucial for the physiological and biochemical processes during plant growth and development, as well as their roles in broader ecological processes [1, 2]. N, a critical component of proteins, plays key roles in various metabolic activities within plants. P is essential for the formation of genetic material and cell structures, while K significantly enhances plant resistance and influences photosynthesis [3]. N and P are identified as the primary limiting nutrients for terrestrial plants [4, 5]. Research by Koerselman, Meuleman, and Güsewell [5, 6] has led to the identification of a threshold ratio of N: P below 14 (or 10) suggests N limitation, while above 16 (or 20) indicates P limitation. K, which is easily leached from the soil, is considered the third major limiting nutrient element after N and P. Venterink’s ertilization experiments in European wetlands introduced a K limitation threshold, proposing that plant growth is limited by K (or jointly by N and K) when N: K exceeds 2.1 and K: P is less than 3.4 [7]. In recent years, more and more studies on plant stoichiometry have been carried out at the macroscopic scale globally, which has weakened the exploration of stoichiometric variation patterns at smaller spatial scales [1]. For the same plant in the long-term monitoring sample plot, its leaf nutrient content is mainly affected by the plant’s growth rhythm, i.e., the plant’s N, P, and K contents will change significantly with the change in the growing season [4]. Leaf N and P contents gradually decline as biomass (growing season) and nutrient resorption (leaf-falling stage) increase. As such, the absorption and utilization of nutrient elements by plants run through the entire growth process, and plants can constantly adjust the allocation and utilization of nutrients in their body, so that each element can play the maximum role in all stages of plant growth and development. Therefore, understanding the nutrient dynamics of plants is essential for revealing their intrinsic growth patterns and their responses and adaptations to the external environment.

Leaf nutrient resorption denotes the transfer of nutrients from senescent leaves to other living tissues before leaf fall occurs [8, 9]. The nutrient concentration control strategy assumes that plants growing in nutrient-poor habitats exhibit higher nutrient resorption efficiency than those in nutrient-rich habitats [10]. Consequently, nutrient resorption efficiency serves as an indicative measure of soil nutrient levels and plant growth status. Plant strategies for coping with nutrient limitation suggest that if a plant is N (P)-limited, it will resorb more N (P) from senescent leaves [11]. As an essential nutrient conservation strategy, nutrient resorption achieves a dual purpose. On the one hand, it diminishes the dependence on soil nutrient supply for plants [12, 13]. On the other hand, the resorbed nutrients can be directly reused by plants, enabling the construction of new tissues at a relatively lower cost compared to nutrient absorption from the soil [11]. The stoichiometric control strategy further underscores the correlation between plant nutrient resorption and source–sink strength, indicating that N and P resorption efficiencies are directly proportional to N: P in green leaves [14]. In general, plant nutrient resorption follows a single strategy [15]. For instance, evergreen plants with long-lived leaves exhibit low nutrient resorption efficiencies despite having relatively low leaf N and P contents and high N: P ratios. Conversely, deciduous plants tend to absorb more nutrients from senescent leaves, allowing green leaves to maintain a high photosynthetic rate and achieve a heightened nutrient resorption efficiency [16]. Like leaf N, P, and N: P, nutrient resorption is influenced by various factors, including plant taxonomy and functional groups, plant age, latitude, temperature, precipitation, and soil fertility [17, 18]. Therefore, exploring the characteristics of plant nutrient resorption is integral to unveiling plant nutrient utilization and survival strategies.

The wild apple trees (*Malus sieversii*) primarily inhabit fragmented region within the Tianshan Mountains, spanning China, Kazakhstan, and Kyrgyzstan. In China, *M. sieversii* is mainly distributed in the Ili Valley, which is considered the last refuge of *M. sieversii* in the world [19]. The wild fruit forest is the Tertiary relict phytocommunity in the mountainous areas in Central Asia and stands as one of the original birthplaces of cultivated fruit trees. As a Tertiary relict species and the precursor to modern cultivated apples [20], *M. sieversii* boasts numerous exceptional properties, rendering it is a precious global wild plant resource [21]. The strategic grafting of *M. sieversii* onto *M. domestica* has yielded hitherto unreported fire blight-resistant germplasm in *M. domestica* [21]. However, in recent decades, the rising temperatures [22] in the Central Asian region have triggered a significant increase in pest numbers [23], posing a severe threat of diseases and pests (mainly caused by *Valsa mali* Miyabe et Yamada and *Agrilus mali* Matsumara) to wild apple trees [24]. Additionally, the continual expansion of cultivated land [25], excessive grazing, and extensive deforestation have led to the reduction of both the distribution range and the population area of wild apples [24]. In a genetic comparison involving 11 wild and domesticated apple varieties from Kazakhstan has underscored a concerning trend: increased human intervention has led to remarkably low genetic diversity among the populations, heightening the risk of extinction for rare plant species [26]. The consequences are evident in the dwindling numbers of natural wild apple trees, with a further reduction in their distribution range, notably between 1300 and 1500 m in the Ili Valley. Challenges such as grazing and mowing have also impeded the survival and regeneration of wild apple seedlings/saplings, resulting in the degradation of wild apple populations in localized areas in Xinjiang [3]. In response to these challenges, *M. sieversii* has been listed as a priority species for biodiversity conservation and is recognized as an essential plant for China’s national secondary protection efforts.

It is well-established that wild apple populations are experiencing severe decline, primarily characterized by a notable increase in dead branch percentage (DBP), with adverse repercussions on their photosynthetic activity. This decline is exacerbated by a decrease in K content, leading to diminished plant resistance, and a combination of multiple factors ultimately culminating in tree mortality [3]. A comprehensive report highlights that, in comparison to relatively healthy wild apple individuals, declining wild apple trees exhibit decreased leaf N and P concentrations, along with a lower N: P ratio [27]. This implies a heightened susceptibility to N limitation. Notably, N-addition experiments conducted on wild apple saplings demonstrate that appropriate N fertilization can effectively stimulate sapling growth, offering a potential avenue for mitigating the decline [28]. A recent study suggests that the growth of declining wild apple trees, particularly those distributed in the narrow valley areas, display a higher sensitivity to interannual environmental changes than changes in elevation [29]. Despite these insights, the dynamics of leaf stoichiometric traits of wild apples, the spatial distribution patterns, and the underlying influencing factors remain unclear, particularly at the sample plot scale.

In this study, we established a permanent sample plot of 100 m × 100 m within a degrading wild fruit forest on the northern slope of Nalati Mountain, a part of Tianshan Mountains, located Xinyuan County, Ili Prefecture, Xinjiang, China. Leaf samples were systematically collected from 25 quadrats, each measuring 20 m × 20 m, during the month of May, July, and October. The leaf N, P, and K concentrations were measured. The study aimed to achieve three main objectives: (1) to comparatively analyze the temporal variability of leaf stoichiometric traits, (2) to explore leaf nutrient resorption efficiencies and elucidate their relationships with nutrients in green and fallen leaves, and (3) to examine the spatial distribution patterns of nutrient traits at the sample plot scale during different growth periods and reveal their influencing factors. The outcomes of this study are anticipated to contribute theoretically by providing a scientific foundation for understanding the mechanism behind the decline of wild apple forests. Moreover, the data generated will offer practical support for the biogeochemical cycle within wild fruit forest ecosystems. In practice, our results can serve as a crucial basis for the conservation and scientific management of wild fruit forest ecosystems.

## Materials and methods

### Study area

The study site (83.60° E, 43.38° N) is in the Ili Botanical Garden, Xinyuan County, Ili Prefecture, Xinjiang, China. The site belongs to the northern slope of the Nalati Mountain, a part of the Tianshan Mountains. This area has a temperate continental climate with a pronounced mountainous terrain, with an average annual temperature of 6–9.3 °C, an average annual precipitation of 260–500 mm, a snowy period of 150 days, a frost-free period of 140–180 days, and an annual sunshine duration of about 2500 h. The Ili Valley is a mountainous valley that opens to the west. The gap to the west is conducive to the entry of moisture from the Caspian Sea and Atlantic Ocean and to the formation of abundant water resources. Wild fruit forest is distributed in the Ili Valley with the ocean climate in the front mountain belt (alt. 1300–1500 m). As a result of the existence of the inversion layer, the mild and humid climatic conditions are conducive to the safe overwintering of *M. sieversii* [30]. *M. sieversii* is the dominant species in the wild fruit forest in Xinjiang, often forming pure forest with high herbaceous diversity. Understory herbaceous plants associated with the forest include *Urtica cannabina*, *U. dioica*, *Phlomis umbrosa*, *Mentha canadensis*, *Agrimonia pilosa*, *Geum aleppicum*, and *Aegopodium kashmiricum*. The soil is derived from loess parent material and is covered by a thick humus layer, endowing it with relatively high fertility.

## Methods

### Field survey and sampling

In August 2016, a permanent sample plot with an area of 100 m × 100 m (i.e., 1 hm^2^) was set up in the centralized distribution area of declining *M. sieversii* on the western side of Ili Botanical Garden. The sample plot was uniformly divided into 25 quadrants with a size of 20 m × 20 m and numbered sequentially (Fig. S1). The plot size and location (elevation range: 1415.4–1446.2 m; Fig. S1) were determined according to the observed distribution range of wild apples here (1300–1500 m). Subsequently, each woody plant with DBH > 1 cm in the sample plot was labeled. *M. sieversii* was the constructive species in this sample plot, and other woody plants were sparse.

Field survey was conducted in May (i.e., spring, the flowering period), July (i.e., summer, the fruiting period), and October (i.e., autumn, the leaf-falling period) in 2017. The survey encompassed a total of 25 quadrats, with two quadrats lacking wild apple trees, and the remaining 23 quadrats were distributed with varying numbers of wild apple trees (Fig. S1). To truly reflect the actual situation of *M. sieversii*, all wild apple trees in the 23 quadrats were sampled. In May and July, one current-year branch was collected from each of the four directions (i.e., east, south, west, and north) in the middle tree canopy (branches extending out of the quadrat were not collected, and branches located in the quadrat area, but the trunks were not in the quadrat were also excluded). All mature and intact leaves on the branches were collected. According to the diagonal method, each quadrat was divided into two halves, and the wild apple leaves in each half of the quadrat were mixed into one sample, resulting in two leaf samples in one quadrat. In October, two litter collectors were positioned in each quadrat, strategically located in two halves of one quadrat. The collectors were located under wild apple trees near the quadrat center to avoid branches not within the same quadrat. The litter collector consisted of a frame of 75 cm × 75 cm in size and a nylon mesh with a 1 mm mesh aperture. The upper surface of the collector was located 1 m vertical from the ground, and the bottom was 25 cm vertical from the ground. Fallen leaves were collected after the litter collector was left for 7 days. Non-wild apple leaves were removed, and all intact fallen leaves from each sample plot were then mixed into one sample.

### Determination of leaf nutrients

Leaf samples were dried in an oven at 70 °C to a constant weight. The dried leaf samples were milled into powder in a vibratory disc mill (MM400, Retsch GmbH Inc., Haan, Germany) and stored in zip bags. The leaf N (mg g^− 1^) concentration was measured using an elemental analyzer (Multi N/C 3100, Analytik Jena AG, Jena, Germany). Leaf P (mg g^− 1^) concentration was measured using the molybdenum–antimony anti spectrophotometric method. Leaf K (mg g^− 1^) concentration was measured by flame spectrophotometry (FP640, Jingke Co., Shanghai, China). The N: P, N: K, and P: K ratios were then calculated.

### Nutrient resorption efficiency

Nutrient resorption efficiency (expressed as *RE*) was calculated using the following equation (Eq. 1):

1 $$RE\; = \;{{({\rm{N}}{{\rm{u}}_\text{leaf}} - {\rm{N}}{{\rm{u}}_\text{litter}})} \over {{\rm{N}}{{\rm{u}}_\text{leaf}}}} \times 100\%$$

where *RE* is the nutrient resorption efficiency, Nu_leaf_ is the leaf nutrient concentration at the peak of plant growth (i.e., in July), and Nu_litter_ is the nutrient concentration of fallen leaves. NRE, PRE, and KRE denote the resorption efficiencies of leaf N, P, and K, respectively.

### Survey of environmental factors

For soil factors, the 5-point sampling method (i.e., plum blossom sampling method) was used to collect a mixed 0–10 cm soil sample in each quadrat. All soil samples were brought back to the laboratory in zip bags, dried naturally in a calm and ventilated place, and sieved for measurement. Soil organic carbon (SOC, g kg^− 1^), total nitrogen (TN, g kg^− 1^), total phosphorus (TP, g kg^− 1^), total potassium (TK, g kg^− 1^), available nitrogen (AN, mg kg^− 1^), available phosphorus (AP, mg kg^− 1^), available potassium (AK, mg kg^− 1^), pH, electrical conductivity (EC, µS cm^− 1^), and total salts (TS, g kg^− 1^) were determined according to standardized methods from Bao [31]. The relevant soil nutrient stoichiometric ratios were then calculated. Soil properties can be found in Table S1.

As for topographic factors, elevation is often used as the main topographic factor for a permanent sample plot in a mountainous area. The slope direction of this large sample plot was northeast. The elevation (1415.4–1446.2 m) of each quadrat had been accurately determined using an Electronic Total-Station (STS-700R, SANDING, China) during the establishment of the sample plot, with a total elevation drop of 30.8 m (Fig. S1).

Biotic factors were also measured in this study. The number of *M. sieversii* in each quadrat was inconsistent, and the plant growth situation also varied. Biotic factors included basal diameter, plant height, plant density, and dead branch percentage (DBP, %) of *M. sieversii*. The basal diameter and plant height were determined using a tape measure and an altimeter pole, respectively. DBP was the percentage of dead branches per plant to the total number of branches per plant; the greater the value of DBP, the smaller the proportion of live branches and the worse the tree’s performance [3, 27, 29].

### Statistics

The mean value of each parameter in the two halves of each quadrat was used to represent the value in one quadrat. The data were checked for normality using Kolmogorov–Smirnov test, and data that did not fit the normal distribution should be logarithmically transformed before being used. One-way ANOVA was used to compare the differences in leaf nutrient traits (including leaf N, P, and K and their stoichiometric ratios and nutrient resorption efficiencies). Levene’s test was used for homogeneity, and Tukey’s test was used for multiple comparisons. The correlations between nutrient resorption efficiencies and leaf N, P, and K and their stoichiometric ratios in July and October were tested via Pearson’s correlation analysis. Statistical analysis and graphing were conducted using SPSS 23.0 (SPSS Inc. Chicago, IL, USA) and Origin 2021 (Originlab Corporation, Northampton, MA, USA), respectively. The allometric relationships among leaf N, P, and K concentrations were described by the power equation *Y* = *βX*^*α*^ (where *X* or *Y* is N, P, or K concentration [here, *Y* vs. *X* indicates N vs. P, N vs. K, and P vs. K, respectively], and *α* is the scaling exponent), which was used to examine covariations in N, P, and K concentrations. The power function was usually log10-transformed. Reduced major axis linear regression was applied to estimate *α* in the scaling function using the “smatr” package in R version 4.2.2 (www.r-project.org). Likelihood ratio tests were applied to investigate the difference between each scaling exponent and 1.0 [32–34].

The spatial variability of *M. sieversii* in the permanent sample plot was characterized using geostatistics: the semivariogram model fitting and mapping. The semivariogram was estimated using the following equation (Eq. 2):

2 $$r\;\left( h \right) = {1 \over {2N\;(h)}}\sum\nolimits_{i = 1}^{N(h)} {[Z(xi) - Z(xi + h)]}$$

where *r*(*h*) is the semivariance at a given distance *h*, Z(*xi*) is the value of the variable *Z* at the *x*_*i*_ location, and *N*(*h*) is the number of pairs of sample points separated by the lag distance *h*. *C*_*0*_ is the nugget variance, and *C* is the asymptote of semivariance *r*(*h*). Structural variance ratios [*C*/(*C*_*0*_ + *C*)] exceeding 75% indicate strong spatial dependence. Ratios from 25 to 75% indicate moderate spatial dependence, and ratios less than 25% indicate weak spatial dependence [35]. Lag distance can also be referred to as effective distance. The range represents the spatial influence range at a certain observational scale, indicating the scale range of spatial autocorrelation variation of the study variable. Its size is constrained by the observational scale. Within the range, the smaller the distance between sample points, the greater the similarity and spatial correlation. When *h* is greater than the range, the spatial correlation of the regionalized variable *Z*(*x*) does not exist. That is, when the distance between a certain point and known points is greater than the range, the data at that point cannot be used for interpolation or extrapolation. Structural variation is non-random variability caused by the spatial arrangement within the study plot. The semivariance, fitting of models to semivariograms, and Kriging mapping were completed using GS + 9.0 (Gamma Design Software, USA).

To further reveal the factors affecting the spatial variation in leaf nutrient traits in the permanent sample plot in different seasons, environmental (i.e., biotic, geographic, and soil variables) covariance analyses were performed in R 4.2.2 (www.r-project.org) using the “vegan”, “tidyverse”, and “Hmisc” packages. Subsequently, environmental factors were randomly selected for removal to avoid the interference of subjective factors. The filtered environmental factors were analyzed for relative contribution in R 4.2.2 (www.r-project.org) using the “hier. Part” package. Correlation analyses between leaf nutrient traits and environmental factors were performed in R 4.2.2 using the “readxl,” “tidyverse,” and “corrplot” packages.

## Results

### Dynamics in leaf stoichiometric traits of wild apple trees in three growth periods

**Fig. 1:**
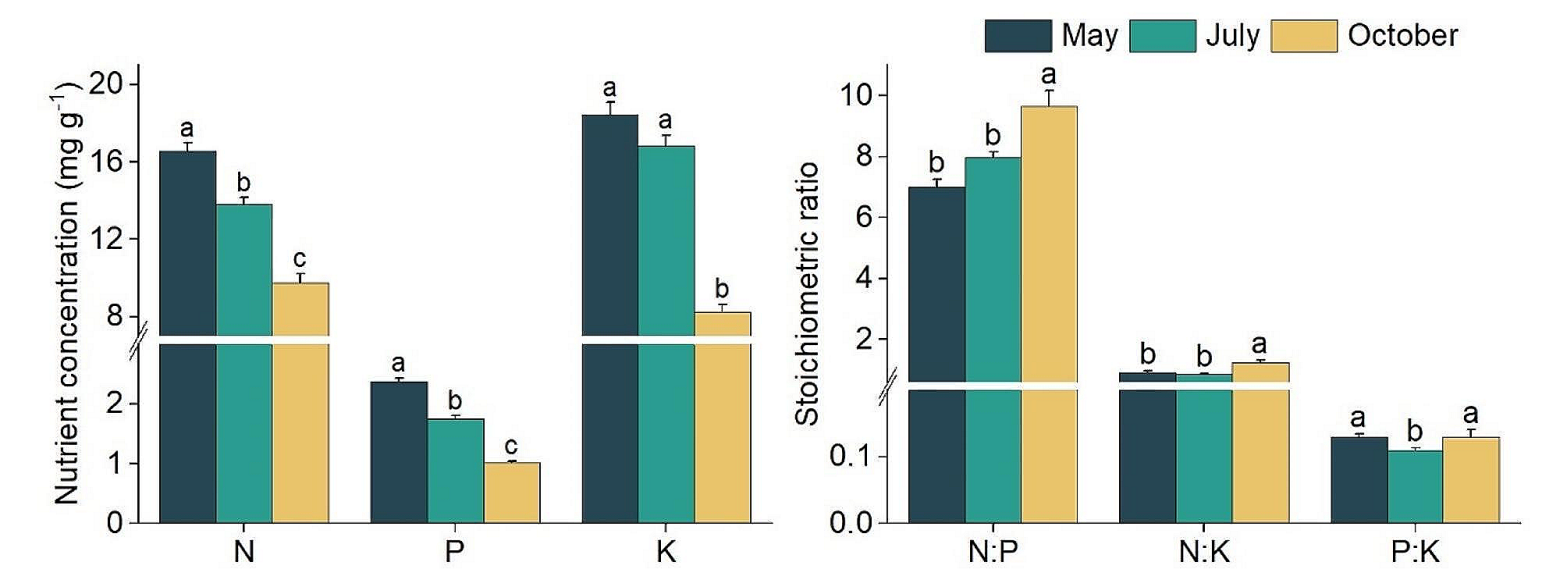
Differences in leaf stoichiometric traits of *M. sieversii* in May, July, and October in the permanent sample plot of a wild fruit forest in Ili Valley, China. Different lowercase letters indicated significant differences among different periods (*P* < 0.05)

The concentrations of N, and P in the leaves of *M. sieversii* displayed significant variations across the three periods. While the K concentrations showed no significant difference between May and July, they were significantly higher than in October. The 3-month mean ranges were 9.74–16.57 mg g^− 1^ for N, 1.02–2.38 mg g^− 1^ for P, and 8.25–18.46 mg g^− 1^ for K. However, all three nutrient parameters significantly decreased with the progression of the growth period (Fig. 1). Significantly, N:P and N: K ratios were markedly higher (*P* < 0.05) in October (9.66 and 1.24, respectively) than in May (7.02 and 0.92, respectively) and July (7.95 and 0.85, respectively). No significant difference was found between May and July. P: K ratio was significantly lower (*P* < 0.05) in July (0.11) than in May (0.13) and October (0.13), although no significant difference was observed between May and October.

In May, we identified significant positive correlations (*P* < 0.001) between leaf N and N: P, N: K, P, and P: K. Conversely, we observed marked negative correlations (*P* < 0.001) between P and N: P and between K and both N: K and P: K. Additionally, we noted considerable positive correlations (*P* < 0.01) between N: P and N: K as well as between N: K and P: K (Fig. S2). In July, we observed significant positive correlations (*P* < 0.001) between leaf N and N: P and N: K, as well as between N: K and P: K. There were also significant negative correlations (*P* < 0.001) between K and N: K and P: K. We found marked negative and positive correlations (*P* < 0.05) between P and N: P and between P and P: K, respectively. In October, we observed positive correlations (*P* < 0.001) between leaf N and N: P and N: K, between N: P and N: K, and between N: K and P: K. Additionally, we identified negative correlations (*P* < 0.001) between leaf K and both N: K and P: K. However, no considerable relationship existed among leaf N, P, and K concentrations at any period.

The pooled data for the three periods revealed that leaf N–P, N–K, and P–K scaling exponents were 1.306, 1.411, and 1.081, respectively (Table 1). Leaf N versus P and N versus K scaling slopes were significantly higher than leaf P–K, that is, they were hyperallometric, hyperallometric, and isometric relationships, respectively. The leaf N concentration decreased more rapidly than leaf P and K during the growth of wild apple. By contrast, a synchronized partitioning rate was found between leaf P and K.

**Table 1:** Allometric scaling exponents and isometric test among leaf N, P, and K concentrations in the permanent sample plot of a wild fruit forest in Ili Valley, China

Y	X	Allometric scaling exponent (a)				Isometric test	
		*R*²	*P*	a		F	*P*
N	P	0.652	0.000	1.306 ± 0.188a		14.060	< 0.001
N	K	0.453	0.000	1.411 ± 0.255a		15.105	< 0.001
P	K	0.724	0.000	1.081 ± 0.139b		1.462	> 0.05

Different lowercase letters indicated significant differences (*P* < 0.05) among scaling exponents. *n* = 69

### Nutrient resorption efficiency of wild apple trees

**Fig. 2:**
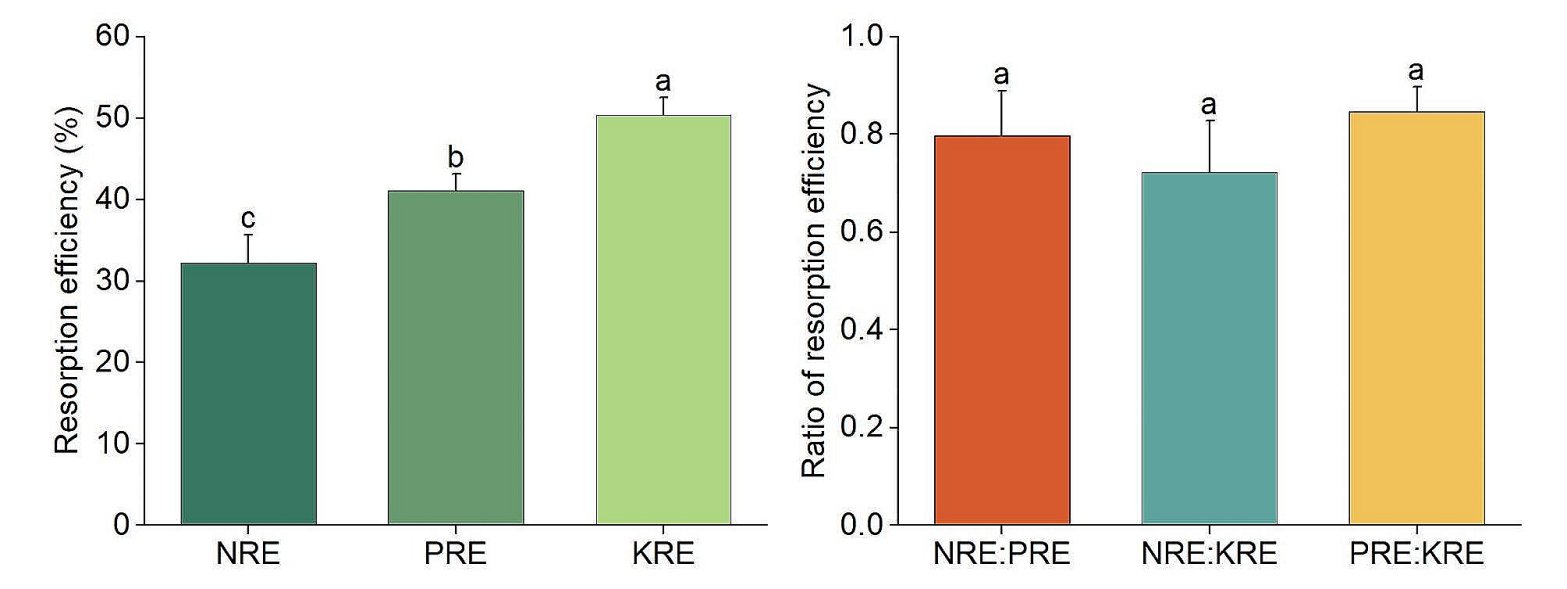
Resorption efficiency of leaf N, P, and K and the stoichiometric ratios of *M. sieversii* in the permanent sample plot of a wild fruit forest in Ili Valley, China

In the permanent wild fruit forest plot, significant differences were identified among the nutrient resorption efficiencies (leaf NRE, PRE, and KRE) of wild apples. The order was KRE (50.38%) > PRE (41.10%) > NRE (32.22%), indicating that *M. sieversii* exhibited the highest demand for K and the lowest for N (Fig. 2). No significant difference were observed among the ratio NRE: PRE, NRE: KRE, and PRE: KRE. In July, PRE was significantly positively correlated with leaf P, and KRE was markedly negatively correlated with leaf N: P. Except for those two cases, no other significant correlation was tested between three nutrient resorption efficiencies and leaf stoichiometric traits. In October, NRE, PRE, and KRE were negatively correlated with N, P, and K in fallen leaves (*r* = − 0.687 to − 0.909), respectively (Fig. S7). In addition, NRE was negatively correlated with N: P and N: K, and KRE was positively correlated with the three stoichiometric ratios of fallen leaves (Table 2). The nutrient resorption efficiencies of wild apples in the permanent plot were primarily controlled by the nutrient concentrations of fallen leaves, rather than green leaves.

**Table 2:** Correlation coefficients between leaf nutrient resorption efficiency of *M. Sieversii* and N, P, and K concentrations in July and October in the permanent sample plot of a wild fruit forest in Ili Valley, China

Period	Parameter	*N*	*P*	K	*N*: *P*	*N*: K	*P*: K
July	NRE	0.187	0.013	−0.312	0.123	0.390	0.340
	PRE	0.211	0.452*	−0.103	−0.134	0.183	0.372
	KRE	−0.288	0.223	0.313	−0.393	−0.420*	−0.201
October	NRE	−0.909**	−0.248	0.174	−0.721**	−0.739**	−0.356
	PRE	−0.199	−0.821**	−0.332	0.281	0.044	−0.267
	KRE	0.405	−0.172	−0.687**	0.482*	0.689**	0.561**

**: *P* < 0.01; *: *P* < 0.05

### Distribution patterns of leaf stoichiometric traits of wild apple trees in three growth periods

The semivariogram models successfully fitted seven out of nine leaf nutrient parameters across the three growth periods, aligning with spherical, exponential, and Gaussian models (Table 3). All seven leaf nutrient parameters showed strong structural variance (structural coefficients of variance were 70.1–97.9%), and leaf N in May showed the highest structural variance. Leaf nutrient parameters showed high spatial dependence, indicating they were dominantly influenced by spatial factors rather than by random factors. The lag distances of seven leaf nutrient parameters varied from 28.5 m to 78.5 m, with leaf K in October having the smallest range, whereas leaf N and P in October exhibited the highest range. For five stoichiometric ratios that could be fitted by the semivariogram model, the structural variance ranged from 2.2 to 18.1% (Table S2). An exception was observed in October, where the structural variance of leaf N: P reached 47.7% (Table S2). This indicated that the spatial variance of leaf stoichiometric ratios was mainly affected by random factors.

**Table 3:** Semivariogram models and parameters of leaf N, P, and K concentrations of *M. Sieversii* in May, July, and October in the permanent sample plot of wild fruit forest in Ili Valley, China

Nutrient	Model	Nugget	Sill	Structural variance (%)	Range (m)	*R*²
N (May)	Spherical	0.001	0.058	97.9	35.7	0.767
K (May)	Exponential	0.016	0.111	86.0	52.5	0.159
N (July)	Spherical	0.002	0.049	96.6	47.0	0.761
P (July)	Exponential	0.005	0.055	90.3	50.4	0.443
N (October)	Gaussian	0.039	0.198	80.4	78.5	0.712
P (October)	Spherical	0.009	0.029	70.1	76.9	0.787
K (October)	Spherical	0.004	0.103	96.0	28.5	0.194

**Fig. 3:**
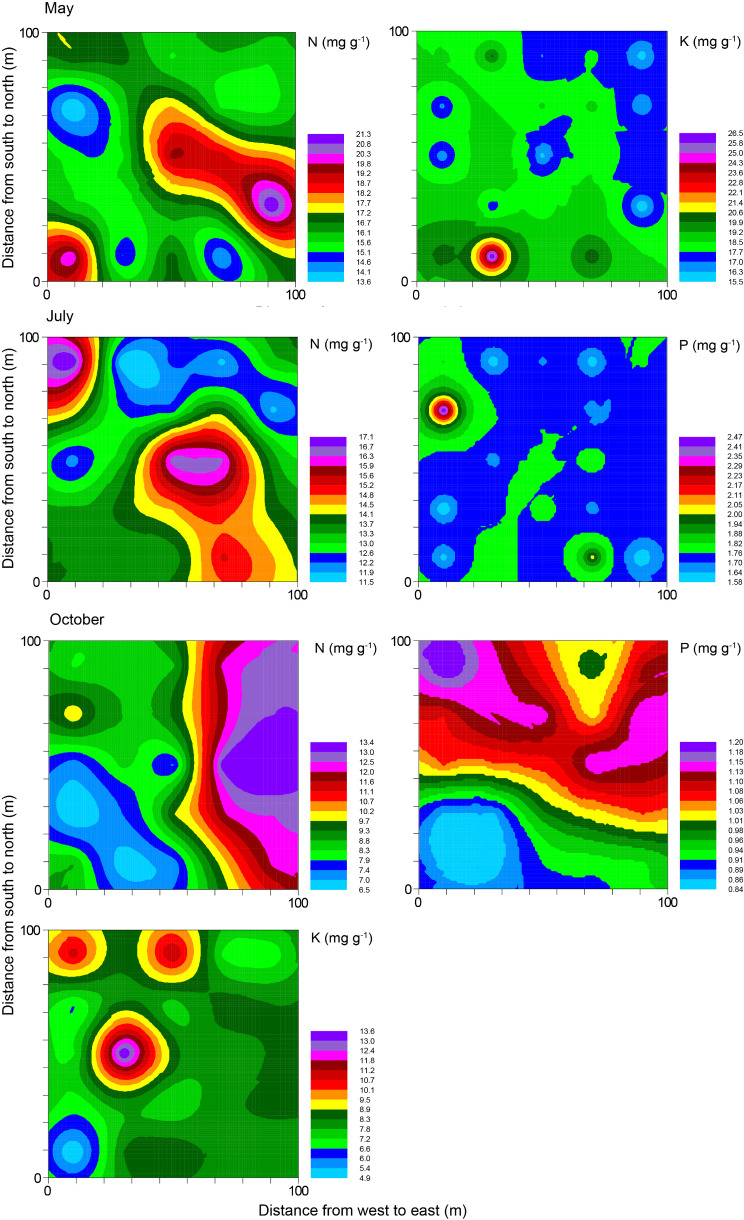
Kriging maps of leaf N, P, and K concentrations of *M. sieversii* in May, July, and October in the permanent sample plot of a wild fruit forest in Ili Valley, China

The kriging interpolation maps vividly portrayed distinct spatial patterns for leaf nutrient concentrations and stoichiometric ratios (Fig. 3 and Figs. S3–S5; Table S2). For instance, leaf P in October showed a high value in the northeast part of the sample plot, but it had a relatively low value in the southwest part of the plot. In contrast, other stoichiometric traits did not exhibit a consistent pattern, and displayed irregular distribution patterns.

### Spatial distribution patterns of leaf nutrient resorption efficiencies of wild apple trees

The semivariogram models for the nutrient resorption efficiencies (NRE, PRE, and KRE) of wild apples were characterized by Gaussian (NRE and PRE) and linear (KRE) models (Table 4). The structural variances of NRE, PRE, and KRE were 66.8%, 50.1%, and 30.2%, respectively. NRE was primarily influenced by structural variance, while KRE was predominantly influenced by random factors, PRE, on the other hand, was influenced by a combination of structural variance and random factors. When considering lag distances, NRE (87.122 m) and KRE (89.4 m) displayed similar values, while PRE exhibited a more substantial lag distance of 137.0 m. The ratios among the three nutrient resorption efficiencies had low structural variance (1.4–44.7%), in which PRE: KRE showed the lowest value (Table S2). In terms of the range (lag distance), NRE: PRE had the highest value of 134.58 m, followed by NRE: KRE (96.3 m), and PRE: KRE had the lowest range at 26.7 m.

**Table 4:** Semivariogram models and parameters of leaf N, P, and K resorption efficiencies of *M. Sieversii* in the permanent sample plot of a wild fruit forest in Ili Valley, China

Variable	Model	Nugget	Sill	Structural variance (%)	Range (m)	*R*²
NRE	Gaussian	108.800	327.400	66.8	87.1	0.933
PRE	Gaussian	0.431	0.864	50.1	137.0	0.652
KRE	Linear	85.978	123.178	30.2	89.4	0.115

**Fig. 4:**
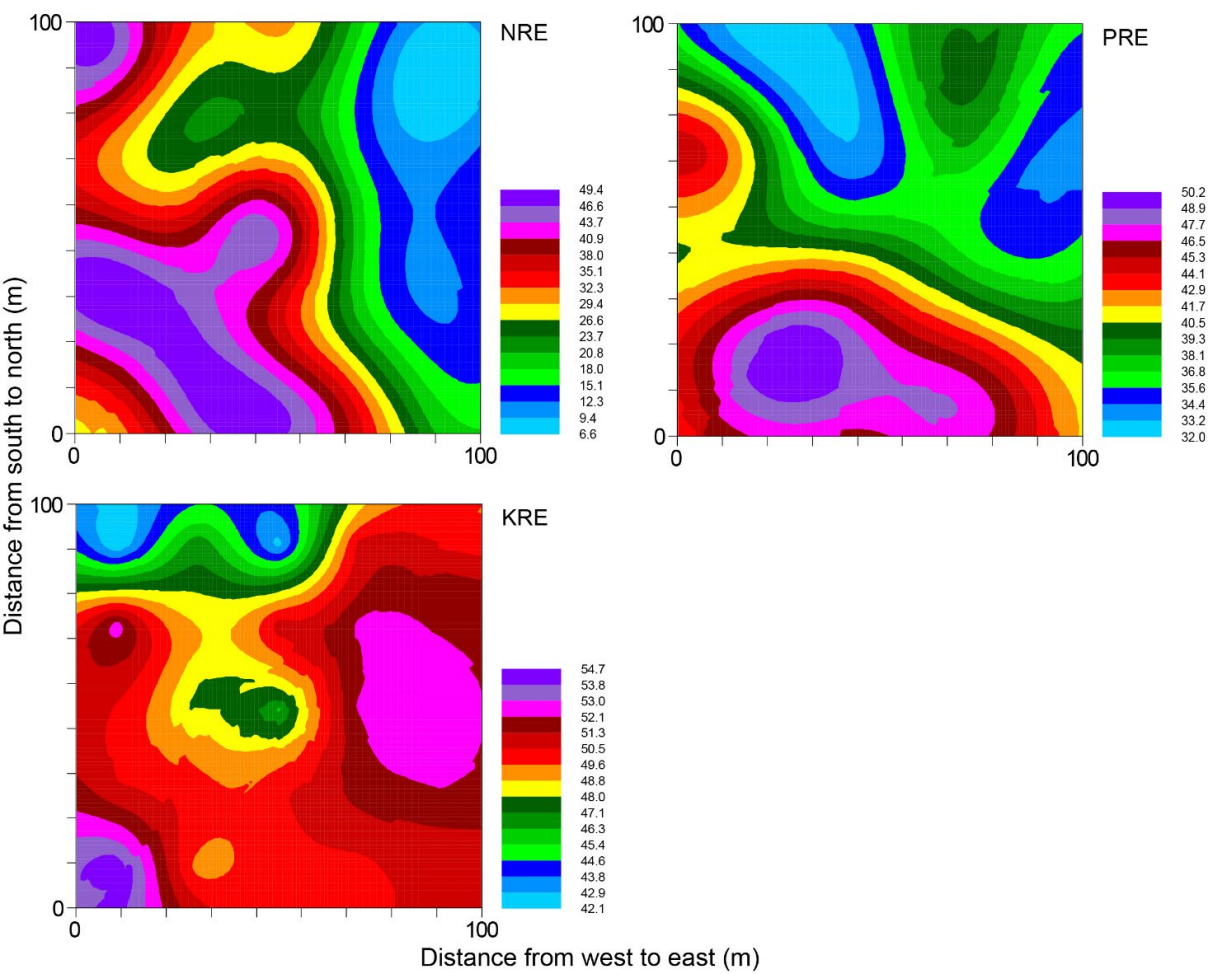
Kriging maps of leaf N, P, and K resorption efficiencies (%) of *M. sieversii* in the permanent sample plot of a wild fruit forest in Ili Valley, China

The kriging interpolation maps indicated that NRE and PRE showed similar spatial patterns, with the highest value in the southwestern plot and the lowest in the northeastern plot (Fig. 4). By contrast, KRE did not show a clear distribution pattern. The spatial patterns of NRE: PRE and NRE: KRE were also similar, both of which showed that the eastern plot had high values, while the western plot had low values. PRE: KRE was poorly fitted and showed an irregular pattern (Fig. S6; Table S2). Therefore, different leaf nutrient resorption efficiency parameters showed varied spatial distribution patterns across different growth periods.

### Influencing factors of leaf nutrient traits of wild apple trees

**Fig. 5:**
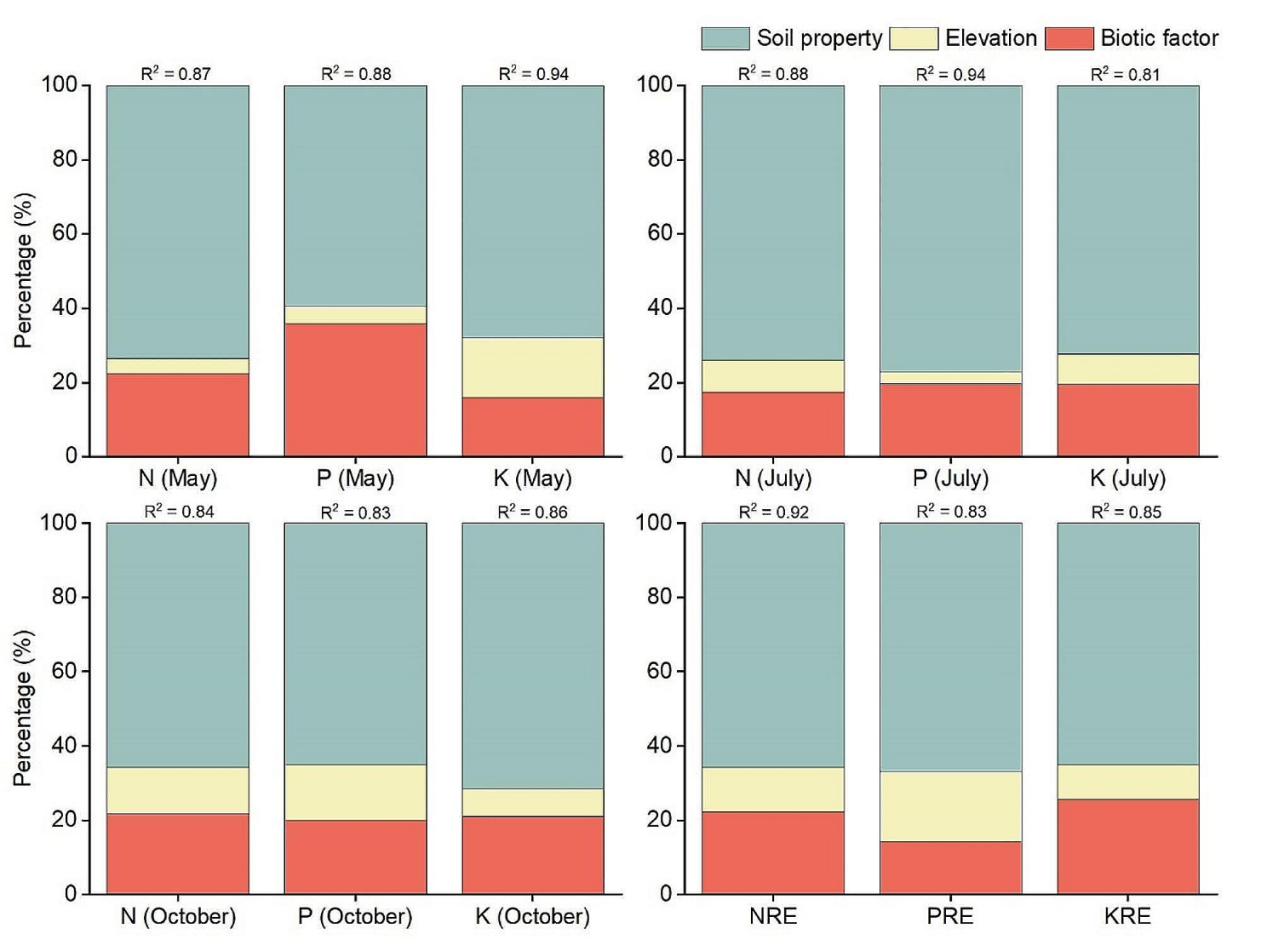
Relative contribution (%) of soil, elevation, and biotic factor to the variations in leaf N, P, and K concentrations in three growth periods and nutrient resorption efficiencies (NRE, PRE, and KRE) of *M. sieversii* in the permanent sample plot of a wild fruit forest in Ili Valley, China

The final *R*^2^ values that explained the variations in leaf nutrients of wild apples reached 0.81–0.94, showing good explanatory powers (Fig. 5; Table S3). Besides, soil property presented the highest relative contribution for all leaf nutrient traits (59.41–77.09%). In May, among the individual soil factors, pH, AP: AK, and SOC: TN had the highest explanatory power to leaf N, P, and K, accounting for 14.21%, 25.1%, and 12.19%, respectively. The biotic factors (basal diameter, plant height, and DBP) collectively contributed significantly to leaf N and P in wild apples, with the relative contribution rates reaching 22.53% and 35.84%, respectively (Fig. 5); however, elevation contributed for a mere 3.92% and 4.77%. Biotic factors and elevation contributed almost the same for leaf K (15.99% and 16.09%, respectively). In July, soil TS, TP, and TP held the highest relative contribution to leaf N, P, and K at 11.91%, 16.95, and 13.85%, respectively. The relative contribute rates (17.34–19.71%) of biotic factors to leaf N, P, and K were all greater than that of elevation (3.2–8.09%). In October, the relative contribution trends for soil properties, elevation, and biotic factors to leaf N, P, and K remained consistent with July (Fig. 5). The relative contribution rates of soil properties, biotic factors, and elevation to leaf N were 65.76%, 21.79%, and 12.45%, respectively. For leaf P, the relative contribution rates of soil properties, and biotic factors, elevation were 65.08%, 19.95%, and 14.96%, respectively. For leaf K, the relative contribution rates of soil properties, and biotic factors, elevation were 71.62%, 21.06%, and 7.32%, respectively. Among the individual soil factors, the relative contribution rates of soil TN: TP to leaf N and P, and soil EC to leaf K showed the largest contribution rates (10.07%, 11.47%, and 15.7%, respectively; Table S3).

In terms of nutrient resorption efficiencies (NRE, PRE, and KRE), soil properties also showed the most significant impact, with relative contribution rates for NRE, PRE, and KRE at 65.67%, 66.93, and 65.12, respectively (Fig. 5). Within soil properties, soil AP: AK, TN: TP, and TN: TP had the greatest relative contribution rates for NRE (28.9%), PRE (10.34%), and KRE (11.08%). Elevation played a smaller role, contributing only 12.11% and 9.35% to NRE and KRE, respectively (Table S3). Biotic factors exhibited a higher relative contribution rate to NRE and KRE (22.21% and 25.53%, respectively), while elevation showed a higher relative contribution to PRE (18.98%). In summary, different leaf nutrient traits of wild apple trees at the sample plot scale were influenced by varied factors in different growth periods, but soil factors contributed largely.

## Discussion

### Leaf N, P, and K stoichiometry of wild apple trees in the permanent sample plot

Plant stoichiometric traits across different months not only provide insight into dynamic changes in nutrient element availability in the environment but also reflect the biological characteristics of plants during various phenological stages [36]. In this study, leaf P content of wild apples showed a decreasing trend from May to October, while the, leaf N: P showed an increasing trend (Fig. 1). This observed pattern aligns with the principles of the Plant Growth Rate Hypothesis (GRH), which posits that a low N: P ratio indicates a high plant growth rate. Specifically, May is the rapid growth period for wild apples, characterized by young fruits, peak levels of N and P in the leaves, and the lowest N: P ratio, indicating a high growth rate [32, 37]. In July, wild apples transition into the mature phase, and the growth rate decreases. Rapid conversion and accumulation of organic matter in the leaves lead to a swift increase in leaf biomass, which dilutes the N and P contents due to the increase in biomass. By October, when the wild apples enter a senescence phase, leaf N and P contents decrease again, and the N: P ratio increases.

Leaf nutrient contents varied largely among different plant species, regions, and ecosystems [34, 38–40]. Research have indicated a linear positive correlation between soil N and leaf N contents [41]. However, the leaf N concentration of wild apples in May (16.57 g kg^− 1^), July (13.82 g kg^− 1^), and October (9.74 g kg^− 1^) in the present study was relatively low. This might be attributed to the specific location of this study in the Ili Valley in the western Tianshan Mountains, where the precipitation is relatively abundant, with an average annual precipitation of 260–500 mm. The increased summer precipitation can result in the leaching of highly mobile available nutrients from the soil. High losses of available N in the soil can lead to a reduction of the N content available for plant growth, thereby causing a decrease in the leaf N content of wild apples [36]. In July, leaf P concentration (1.75 mg g^− 1^) of wild apples was relatively high in other known plant species [33, 38, 39]. Despite China having relatively low soil P content (0.56 g kg^− 1^) [42], P content gradually increasing from wetter areas to arid and semi-arid regions [38], and the P content in plants changes accordingly. Moreover, leaf K contents in May and July (18.46 and 16.8 g kg^− 1^, respectively) surpassed the leaf K content (13.49 g kg^− 1^) observed in a study involving 654 plant species in the north–south transect in eastern China [38]. Research suggests that K can mitigate the incidence rate of plant disease caused by fungal attacks, and a close correlation exists between K content and the severity of rot disease occurrence [43, 44]. An investigation into the relationship between the DBP of *M. sieversii* and leaf K content has found that the leaf K content gradually decreases as DBP increases. Unhealthy wild apples have low K content in their photosynthetic and reproductive organs. And leaf K content and its stoichiometric ratios exhibit significant differences among different categories of DBP [3]. These findings underscore the positive impacts of increased K content on enhancing resistance and promoting the overall growth of wild apples.

The spatial distribution pattern of nutrients generally results from the combined effects of structural factors (e.g., climate, soil parent material, topography, and soil type) and random factors (e.g., fertilization, cultivation practices, and planting systems). Structural factors generally lead to strong spatial heterogeneity and spatial correlation of leaf nutrients, while random factors can diminish spatial correlation and lead to homogenization [35]. In this study, except for leaf P content of wild apple in October (which showed moderate structural variability), all nutrient traits showed strong spatial autocorrelation (Table 3). However, with significant differences in lag distances (28.5–78.5 m), leaf nutrient traits of wild apples in different growth stages showed irregular distribution patterns, such as patchy or lumpy, reflecting the apparent spatial heterogeneity of nutrient traits of wild apples at the sample plot scale (small spatial scale).

### Nutrient resorption of wild apple trees in the permanent sample plot

Nutrient resorption efficiency indicates plant nutrient utilization characteristics and indirectly signifies nutrient status in the soil. The resorption efficiency of N, P, and K in terrestrial plants at the global scale was reported to be 62.1%, 64.9%, and 70.1%, respectively [45, 46], all of which were higher than the values observed in the present study. This discrepancy might be related to the limited nutrient conservation and utilization capacity of declining wild apples, as well as potentially influence of the nutrient resorption strategy of themselves. When the leaf stoichiometric ratio of plants falls within the threshold of nutrient element limitations, they adeptly adjust their nutrient absorption strategies in response to subtle deficiencies in essential elements [12, 15]. For instance, when plants are limited by N (or P), they tend to absorb more N (or P) from senescing leaves, leading to an increase in NRE (or PRE) [40, 47]. The Relative Nutrient Resorption Hypothesis suggests that there are interactions between different nutrient elements during nutrient resorption [11], implying that if a plant is in a balanced growth state, its nutrient resorption will also be in equilibrium, and vice versa.

In the present study, leaf P content and PRE in July were significantly related, showing higher PRE with increased P in green leaves (Fig. S7). However, in October, significant correlations were found between N, P, and K concentrations and their respective resorption efficiencies, with the resorption efficiencies decreasing as the concentrations of leaf N, P, and K increase (Fig. S7). These findings suggested that nutrient resorption efficiency in the sample plot is predominantly influenced by the nutrient levels in fallen leaves. Woody plants in numerous regions exhibited higher NRE (56.8%) and PRE (56.3%) than those observed in this study [11, 48, 49]. The distinctive nature of wild fruit forests, the only deciduous broadleaf forests in the arid Central Asia, and the specific climate and the forest characteristics may have contributed to this unique nutrient resorption strategy. Moreover, in Karst regions with N-limitation and non-Karst regions with P-limitation, their NRE and PRE are higher than those observed in wild apples, respectively [12]. This difference may be related to the soil nutrient content, as the soil TN in Karst regions (4.31 mg kg^− 1^) and soil TP in non-karst regions (0.17 mg kg^− 1^) are both lower than that in wild fruit forests (soil TN: 9.60 mg kg^− 1^; soil TP: 1.56 mg kg^− 1^, Table S1). The reduced nutrients availability for plant absorption from the soil could lead to increased nutrient resorption. For KRE, differences were also observed between this study and other regions [48, 49]. *M. sieversii* is susceptible to rot diseases and *Agrilus mali* infestations. Trees with severe growth decline experience a significant reduction in NRE, a declining trend in PRE, and an increasing trend in KRE [3]. This implies that severe growth decline has a significant negative impact on nutrient resorption efficiency, aligning with the actual growth of wild apples in our permanent sample plot [27]. Severe declines in wild apples markedly deplete K reserves at various growth stages to improve their stress resistance, resulting in an increasing need to reuse large amounts of K [3]. In addition, NRE, PRE, and KRE all exhibited moderate structural variation (Table 4), with relatively large lag distances. All of them displayed irregular distribution patterns, i.e., spatial heterogeneity.

Now, the nutrient limitation types and limiting strength of wild apples in the sample plot can be discussed. Wild apples in July had a leaf N: P of approximately eight, far below the threshold of 16. When the ratios of NRE: PRE, NRE: KRE, and PRE: KRE are all less than 1, it suggests that N, P, and K are all limiting factors for growth [50]. In addition, compared with May, the decline rates of nutrient elements followed the order of N (30%) < P (42%) < K (51%), while the nutrient resorption efficiency showed an order of KRE > PRE > NRE. This suggested that the nutrient limitation sequence for wild apples was K (enhancing resistance) > P (controlling the growth rate) > N (regulating physiological activity). Furthermore, some studies suggest that NRE and PRE are positively correlated with the N: P ratio in green leaves [14]. However, in this study, the nutrient concentrations in senesced leaves primarily regulated NRE, PRE, and KRE. This result highlighted the unique resorption strategy employed by wild apples.

### Factors influencing the nutrient traits of wild apple trees in the permanent sample plot

Under natural conditions, the well-being of plants is intricately shaped by the interplay of various factors rather than a singular influence. For instance, wild apple trees can be affected by *A. mali*, *V. mali*, grazing, and logging. Excessive disturbance can lead to the death of branches and, in severe cases, the death of the entire plant. The death of branches poses various hazards to *M. sieversii*: (1) C starvation: branch death leads to a decrease in photosynthetic product accumulation, resulting in C starvation throughout the plant, which may accelerate plant death; (2) nutrient recycling disruption: the dying branches cannot recycle nutrients from senesced leaves to other active tissues, resulting in the lack of nutrients; and (3) reduced fruit production: branch death can decrease the fruiting rate, affecting the reproductive capability of wild apple trees. This decline can result in difficulties in the survival and regeneration of wild apple seedlings. These implications highlight the complex interactions between various factors that influence the health and growth of *M. sieversii* [40]. The DBP had the most significant relative contribution to leaf P content in May, leaf N content in October, and KRE of *M. sieversii* (Table S3). This result confirmed the effect of plant growth on its own nutrient status. The body size of plant individuals (reflected by plant height and basal diameter) is influenced by the environmental conditions of their location and their age, and environmental conditions are significantly correlated with age [49]. However, in the present study, no significant relationship was found between DBP and the body size of *M. sieversii*. The plant height had the highest relative contribution to leaf N and K in May, leaf N and K in July, K in October, and PRE of *M. sieversii* (Table S3). Basal diameter showed the highest relative contribution to the leaf P element in July and NRE of *M. sieversii* (Table S3). These results indicated that leaf nutrient traits were more influenced by plant height than by other factors.

Plant growth and development are influenced by habitat heterogeneity. In high mountain environments, changes in elevation can affect climate conditions, thereby impacting plant growth, development, and nutrient stoichiometry [51]. Plants adapt to the changing external climate conditions by adjusting their physiological states and nutrient allocation strategies. For instance, as elevation increases, temperatures gradually decrease, promoting plants to enhance their leaf carboxylation capacity in response to the inhibitory effects of low temperatures on enzymes within their bodies. This increase in carboxylation capacity can lead to high leaf N contents [52]. As elevation increases, the trend in the leaf P content is opposite to that of leaf N. With rising elevation, the soil available P content significantly decreases. Consequently, plants have reduced access to external sources of P. Moreover, because of the lowered temperatures at high elevations, enzyme activity within plants diminishes, leading to reduced growth rates and decreased demand for P [53, 54]. This phenomenon ultimately results in a decline in leaf P content. As a result, plants require more P and less N to be resorbed from the leaves to construct new tissues. This finding was consistent with previous studies on *Picea crassifolia* and *Potentilla fruticosa* [55, 56]. Although the elevation difference of the sample plot in this study was not significant (ranging from 1415.4 m to 1446.2 m), elevation changes could still have a specific impact on plants. The elevation could indirectly affect plant growth by influencing light, temperature, precipitation, soil property, and other environmental factors. These minor changes could also influence plants’ physiological processes and growth dynamics [57]. In this study, elevation generally contributed little to the variation in leaf nutrients; the contribution to leaf K in May (16.09%), and N (12.45%) and P (14.96) in July, and NRE (12.11%) and PRE (18.98%) was slightly higher than its contribution to leaf N and P (3.92% and 4.77%) in May and leaf N, P, and K in July (3.2–8.68%; Table S3). Despite that, the growth of wild apples is susceptible to elevation, which explained the narrow distribution range of wild apples, displaying a belt-like horizontal distribution in several areas along the northern slope of the western Tianshan Mountains [29].

Leaf nutrient contents generally exhibit a positive correlation with soil nutrient contents and the stoichiometric ratios, making plant leaf stoichiometry a valuable indicator of soil nutrient status [58]. In this study, significant correlations were observed between soil AP: AK and leaf P in May as well as NRE, with relative contributions of 25.1% and 28.9%, respectively (Table S3). A significant correlation was observed between soil TP and leaf N and K in July, with relative contributions of 16.96% and 13.85%, respectively (Table S3). Soil TP showed a significant correlation with leaf P in October (Fig. S8), with a relative contribution reaching 13.12% (Table S3). A comprehensive analysis indicated that the relative contribution of soil AP was higher than that of soil TP, suggesting that soil available nutrients had a significant direct effect to plant nutrient content. Moreover, soil nutrient stoichiometric ratios also play crucial rule in plant leaf stoichiometry. Our results showed that when the soil stoichiometric ratios (SOC: TN, TN: TP, and AP: AK) were retained in the models, the *R*^2^ values increased to 0.81–0.94, and AP: AK had the highest interpretation of leaf P in May and NRE, indicating that the soil stoichiometric ratio of the sample plot was an important factor affecting the leaf nutrients of wild apple together with other soil factors. In conclusion, the variation in leaf nutrient traits of wild apples at the sample plot scale was not only affected by the growth period, but the spatial heterogeneity of leaf nutrient traits in different growth periods was also affected by soil properties, elevation, and biotic factors of the plants themselves.

In addition, fine roots are an important link in nutrient turnover in plant-soil systems. Roots are important for anchorage, water uptake, and acquiring mineral nutrients from the soil thereby, playing an important role in the growth and health of plants [59]. Results make it clear that studies of roots and above-ground tree parts are equally important when attempting to assess the vitality of forest trees as a whole [60]. In terrestrial ecosystems, the effects of fine roots on nutrient cycling operate through two key pathways, namely, the fine root absorption of nutrients from the soil for plant growth and the fine root mortality, decomposition, and related rhizosphere processes that return nutrients to the soil [61]. The effect of fine roots on plant functional processes is attributed to changes in physiological characteristics such as growth, morphology, and nutrients, which determine the response of plants to environmental factors and their impact on ecosystems [62]. For instance, an increase in the roots’ absorptive surface of ryegrass, due to the presence of root hairs, enhances the uptake of both P and K from soil [63]. In forests, the amount of C and nutrients returned to the soil from fine root turnover may equal or exceed that from leaf litter [64]. Therefore, soil properties and root structure are the major factors determining the efficiency of nutrient acquisition by plants. The root system is highly plastic, responding to variations in P availability through changes in root morphology, architecture, exudation, and interactions with soil microbes [65]. In forest ecosystems, plants with different functional groups may have significantly different fine-root nutrient strategies, e.g., shallow-rooted plants develop longer and more abundant root hairs, facilitating nutrient uptake, particularly for P [66], reflecting the differentiation of the fine root plasticity and function of plants with different functional groups. It’s revealed that the N: P ratios of both fine roots and leaves decrease with latitude on a global scale. However, at the sample plot scale of wild apple forest, it is unclear what is the relationship between the stoichiometric pattern of fine roots of different components and the nutrients of wild apples. Therefore, this will be one of the focuses of our future research.

## Conclusions

We present the first report on the temporal dynamics in leaf N, P, and K stoichiometry, spatial distribution patterns of leaf stoichiometry and nutrient resorption efficiency, and elemental allometric scaling exponent of wild apples across different growth periods at the sample plot scale in the Tianshan Mountains. Leaf N, P, and K concentrations showed a decreasing trend in May, July, and October. Leaf stoichiometric ratios displayed different trends in various growth periods (i.e., N: P and N: K increased, whereas P: K reduced first and then increased). These changes in the nutrient contents were consistent with the Plant Growth Rate Hypothesis (GRH). Leaf N had a faster decline rate than P and K over the growth stages, while the relationship between P and K remained isometric. Nutrient resorption efficiency followed the order of NRE < PRE < KRE, all of which were mainly influenced by the elemental contents of fallen leaves. A strong spatial dependence of leaf nutrients was observed in each growth period, with a low contribution from random factors. While the three nutrient resorption efficiencies were moderately spatially dependent, with a clear role for random factors. Leaf nutrient traits showed irregular distribution patterns and no consistency across the three growth periods. Leaf nutrients and nutrient resorption efficiencies in each period were simultaneously affected by soil, elevation, and plant growth status, with soil properties having a more significant influence (59.41–77.09%). A comprehensive analysis involving nutrient thresholds, stoichiometric ratio thresholds, and stoichiometric scaling exponent suggested that the order of nutrient limiting strength was K > P > N for wild apple growth. Because wild apples play a crucial role in preserving the original genes of apples on a global scale, and the wild fruit forests are regarded as natural repositories of genetic diversity for various fruit tree resources worldwide. This research also deepens the study of environmental adaptability and nutrient utilization strategies of degraded wild fruit forests at the plot scale, providing a scientific basis for the future scientific management of the wild fruit forests.

### Electronic supplementary material

Below is the link to the electronic supplementary material.

Supplementary Material 1

Supplementary Material 2

## Data Availability

The original contributions presented in the study are included in the article/supplementary material. Further inquiries can be directed to the corresponding author.
