# Supplementary material for: Spatiotemporal patterns of leaf nutrients of wild apples in a wild fruit forest plot in the Ili Valley, China
Source: BMC Plant Biol. 2024 Jul 18;24:684. doi: 10.1186/s12870-024-05417-6 (PMC11256650; doi:10.1186/s12870-024-05417-6)
Supplement: Supplementary file 2 — Supplementary Material 2 [file 12870_2024_5417_MOESM2_ESM.pdf]

Supplementary material

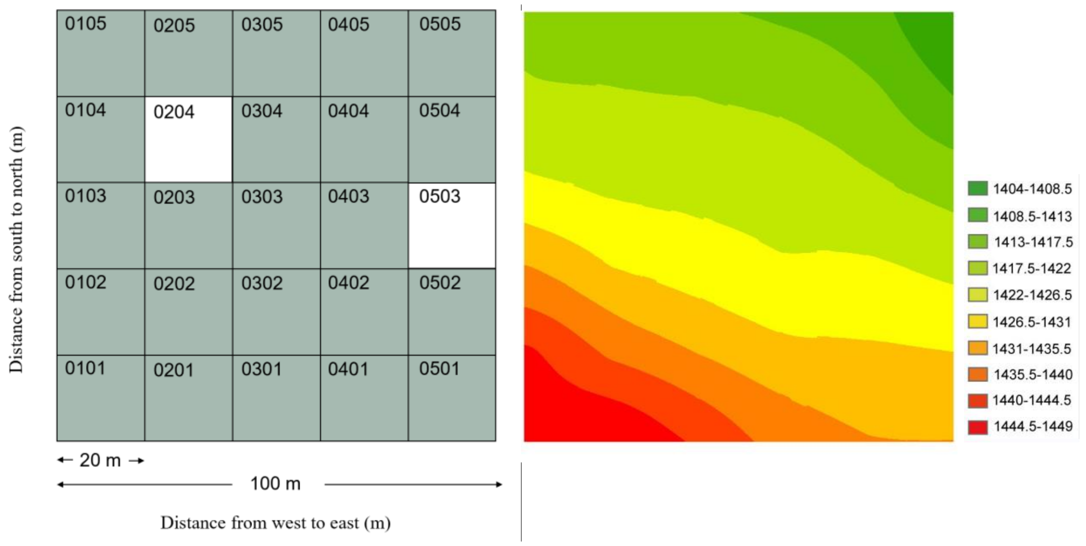

Fig. S1 Schematic diagram of plot division and elevation in the permanent sample plot of a wild fruit forest in Ili Valley, China. No wild apple trees existed in the two quadrats (0204 and 0503).

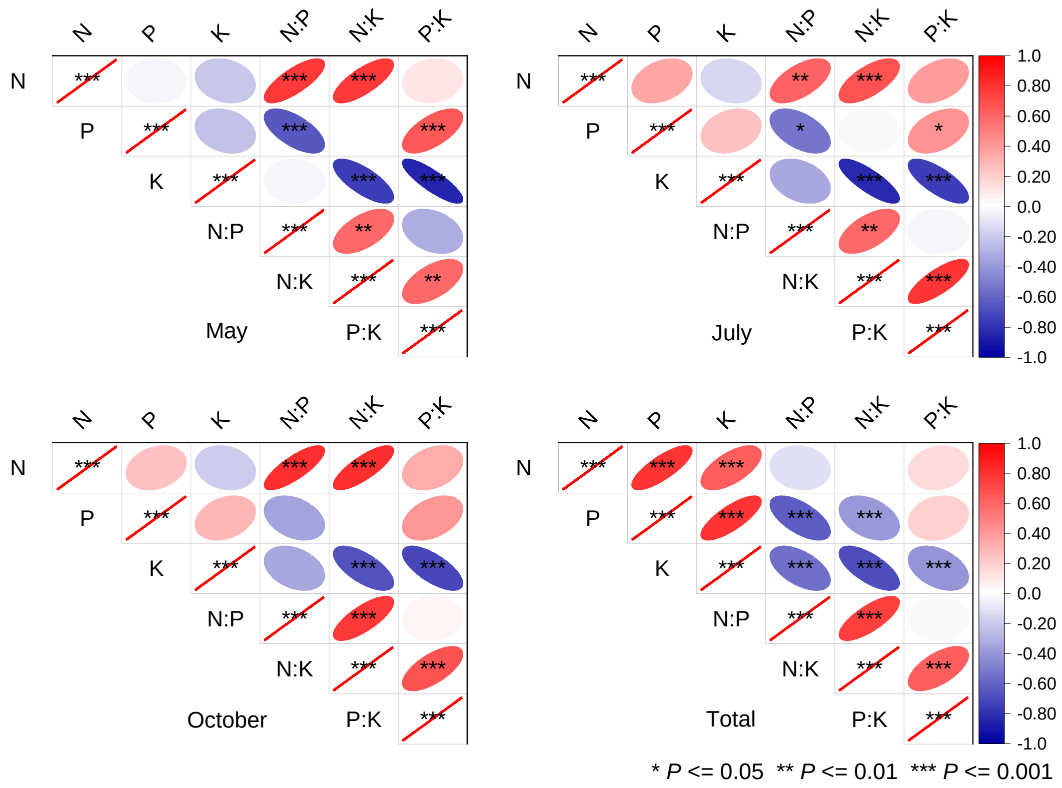

Fig. S2 Correlations among leaf stoichiometric characteristics of N, P, and K of *M. sieversii* in May, July, and October in the permanent sample plot of a wild fruit forest in Ili Valley, China

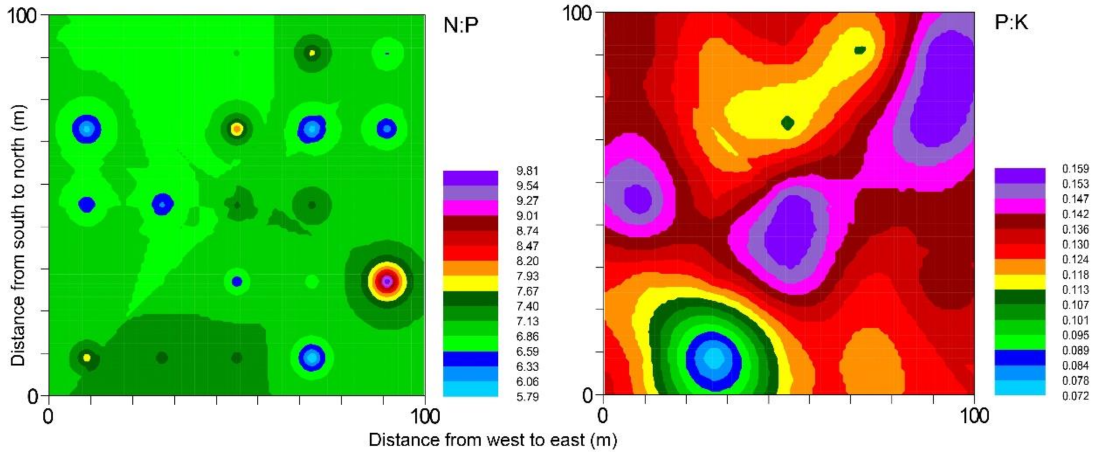

Fig. S3 Kriging maps of leaf N:P and P:K of *M. sieversii* in May in the permanent sample plot of a wild fruit forest in Ili Valley, China

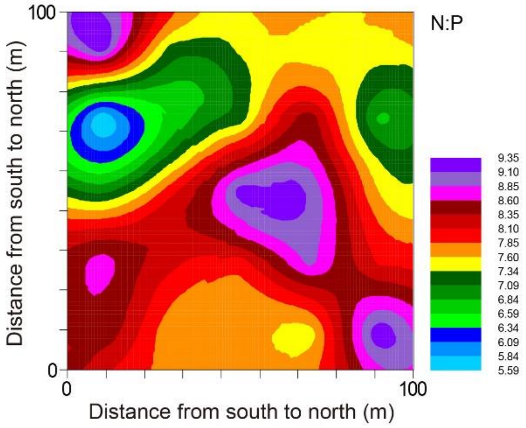

Fig. S4 Kriging map of leaf N:P of *M. sieversii* in July in the permanent sample plot of a wild fruit forest in Ili Valley, China

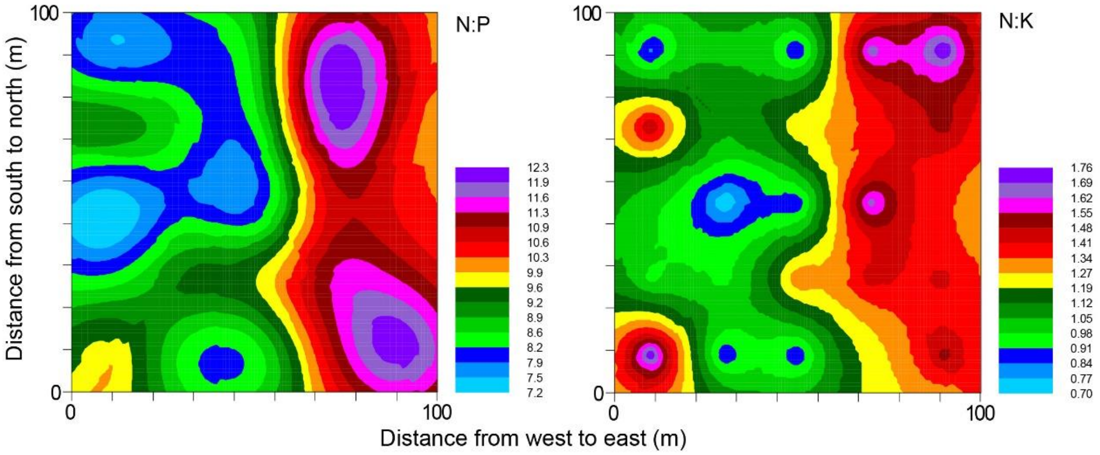

Fig. S5 Kriging maps of leaf N:P and N:K of *M. sieversii* in October in the permanent sample plot of a wild fruit forest in Ili Valley, China

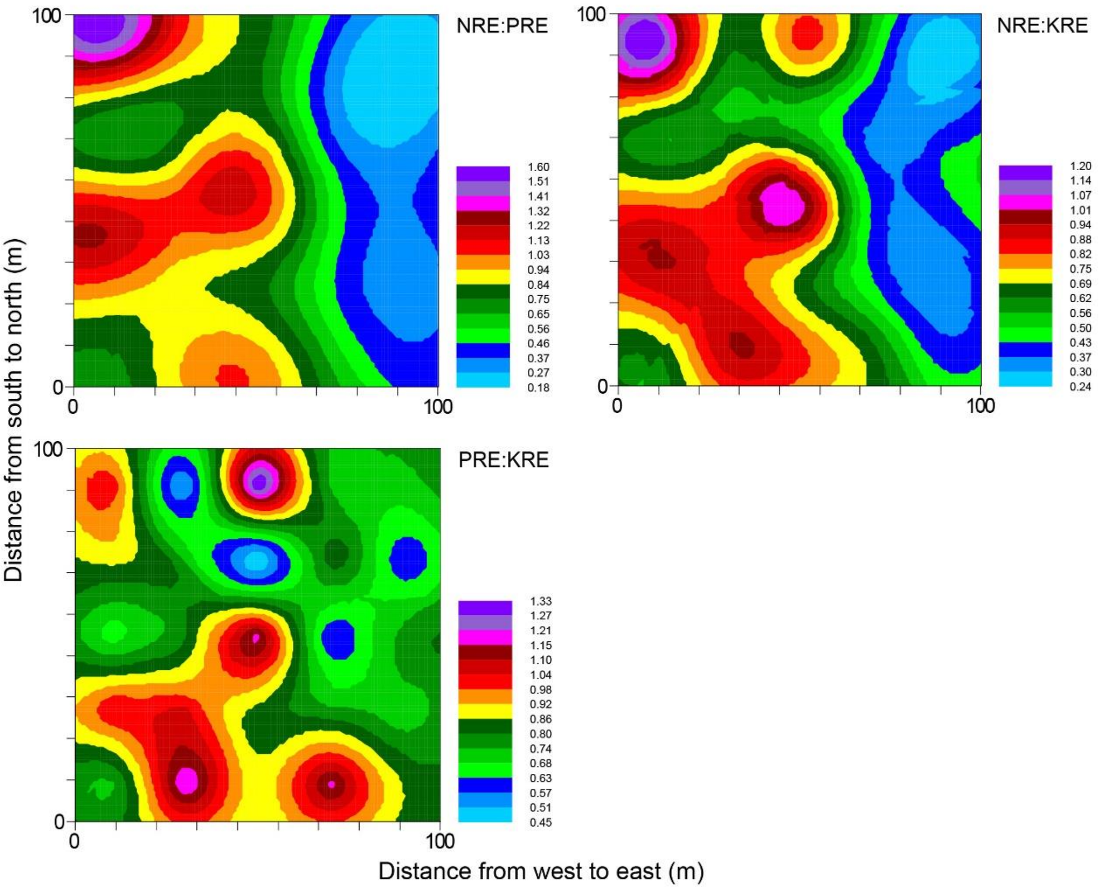

Fig. S6 Kriging maps of nutrient resorption efficiencies (NRE:PRE, NRE:KRE, and PRE:KRE) of *M. sieversii* in the permanent sample plot of a wild fruit forest in Ili Valley, China

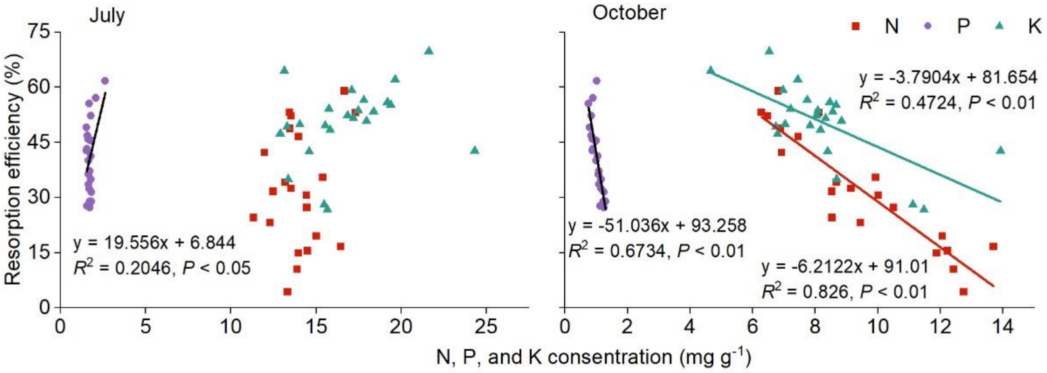

Fig. S7 Relationships between leaf nutrient resorption efficiency of *M. sieversii* and N, P and K concentrations in July and October in the permanent sample plot of wild fruit forest in Ili Valley, China

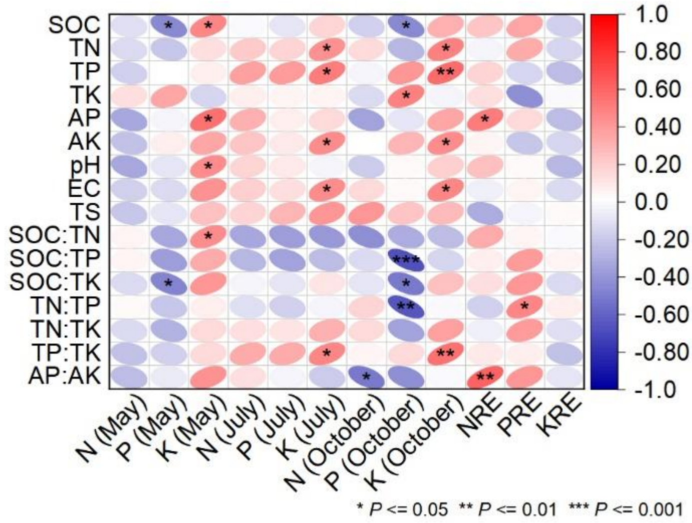

Fig. S8 Correlations between leaf N, P, K, and resorption efficiencies of *M. sieversii* and soil properties in May, July, and October in the permanent sample plot of a wild fruit forest in Ili Valley, China

Table S1 Soil properties in the permanent sample plot of a wild fruit forest in Ili Valley, China

Parameter	SOC ($g\ kg^{-1}$)	TN ($g\ kg^{-1}$)	TP ($g\ kg^{-1}$)	TK ($g\ kg^{-1}$)	AP ($mg\ kg^{-1}$)	AK ($mg\ kg^{-1}$)	pH	EC ($\mu S\ cm^{-1}$)
Mean	102.27	9.60	1.56	19.03	13.29	576.77	6.97	197.58
SE	1.60	0.16	0.02	0.13	0.99	27.15	0.03	5.32
CV (%)	7.83	8.19	7.22	3.29	37.21	23.54	2.19	13.46
**Parameter**	**TS ($g\ kg^{-1}$)**	**SOC:TN**	**SOC:TP**	**SOC:TK**	**TN:TP**	**TN:TK**	**TP:TK**	**AP:AK**
Mean	1.58	10.67	65.52	5.39	6.14	0.51	0.08	0.02
SE	0.05	0.11	1.04	0.11	0.08	0.01	0.001	0.001
CV (%)	14.72	4.97	7.95	10.16	6.32	10.58	7.80	26.04

SOC, TN, TP, TK, AP, AK, pH, EC, and TS represented soil organic carbon, total nitrogen, total phosphorus, total potassium, available phosphorus, available potassium, soil pH, soil electrical conductivity, and total salt content, respectively.

Table S2 Semi-variogram models and parameters of leaf stoichometric ratios of *M. sieversii* in May, July, and October and ratios of nutrient resorption efficiency in the permanent sample plot of a wild fruit forest in Ili Valley, China

Variable	Model	Nugget	Sill	Structural variance (%)	Range (m)	R^2
N:P (May)	Exponential	0.003	0.044	7.7	28.500	0.655
P:K (May)	Spherical	0.000	0.001	2.2	35.800	0.460
N:P (July)	Spherical	0.001	0.031	2.9	40.500	0.672
N:P (October)	Gaussian	0.094	0.198	47.7	94.743	0.917
N:K (October)	Exponential	0.008	0.044	18.1	88.800	0.820
NRE:PRE	Gaussian	0.022	0.115	18.8	134.580	0.892
NRE:KRE	Gaussian	0.047	0.104	44.7	96.302	0.661
PRE:KRE	Spherical	0.000	0.018	1.4	26.700	0.102

Table S3 Relative contribution (%) on leaf N, P, K, and resorption efficiency of *M. sieversii* in the permanent sample plot of a wild fruit forest in Ili Valley, China

Parameter	Biotic factor			Geography	Soil property								
	BD	H	DBP	Elevation	pH	EC	TS	TP	TK	AK	SOC:TN	TN:TP	AP:AK
N (May)	4.34	7.54	10.65	3.92	14.21	5.27	8.03	6.63	8.58	8.41	9.26	8.84	4.32
P (May)	24.24	6.68	4.92	4.77	6.42	1.93	3.45	2.13	7.79	2.32	5.61	4.66	25.10
K (May)	6.52	5.40	4.07	16.09	5.81	11.00	7.63	4.28	7.12	8.03	12.19	2.82	9.04
N (July)	4.21	6.21	6.92	8.68	5.69	6.03	11.91	9.01	7.93	6.41	11.29	9.34	6.38
P (July)	5.56	4.54	9.61	3.20	3.14	5.16	12.81	16.95	6.88	4.80	14.65	7.00	5.70
K (July)	5.28	7.89	6.42	8.09	3.97	11.75	7.73	13.85	6.10	10.65	6.57	4.83	6.87
N (October)	9.09	6.49	6.21	12.45	6.98	5.66	9.51	6.29	5.51	5.95	9.93	10.07	5.86
P (October)	6.62	5.50	7.83	14.96	5.55	7.41	4.71	6.91	7.52	5.77	7.88	11.47	7.86
K (October)	4.99	11.70	4.37	7.32	4.32	15.70	4.95	13.12	6.84	10.02	6.28	7.53	2.86
NRE	8.78	7.84	5.59	12.11	7.15	3.48	7.54	3.30	4.32	3.61	3.16	4.21	28.90
PRE	4.17	3.53	6.39	18.98	3.18	4.18	7.99	7.73	6.40	8.94	8.33	10.34	9.84
KRE	6.89	10.96	7.68	9.35	6.28	5.11	4.91	10.39	9.66	6.83	6.47	11.08	4.39

BD: basal diameter; H: plant height; DBP: dead branch percentage. SOC, TN, TP, TK, AP, AK, pH, EC, and TS represented soil organic carbon, total nitrogen, total phosphorus, total potassium, available phosphorus, available potassium, soil pH, soil electrical conductivity, and total salt content, respectively.
